# Improving in vitro Gastrointestinal Stability of Phlorotannins From Food Grade *Fucus vesiculosus* Extracts Using Cyclodextrins

**DOI:** 10.1111/1750-3841.70830

**Published:** 2026-01-13

**Authors:** Sofia F. Reis, Marcelo D. Catarino, Susana S. Braga, Manuel A. Coimbra, Artur M. S. Silva, Susana M. Cardoso

**Affiliations:** ^1^ LAQV‐REQUIMTE, Department of Chemistry University of Aveiro Aveiro Portugal

**Keywords:** brown seaweed, cyclodextrins, microwave assisted extraction, phlorotannins, polysaccharides

## Abstract

Phlorotannins have gained significant attention for their potential bioactive and functional properties. However, these compounds are highly sensitive to gastrointestinal digestion, which limits their bioavailability and consequently the in vivo efficacy.

To address this limitation, this study explored the use of various concentrations of γ‐ and β‐cyclodextrins in the aqueous microwave‐assisted extraction of phlorotannins from *Fucus vesiculosus* and submitted the resulting extracts to an in vitro gastrointestinal digestion protocol.

While higher cyclodextrin concentrations resulted in an increase in extraction yields (from 47% to approximately 55% for both cyclodextrins), the opposite was observed for the total phlorotannin content. Notably, higher cyclodextrin concentrations markedly improved phlorotannin stability across the different stages of gastrointestinal digestion. Specifically, β‐cyclodextrin exhibited the highest protective effect, preventing phlorotannin degradation almost completely in both oral and gastric phases, and retaining approximately 35% (approximately 1.0 mg/g extract) of the initial content (approximately 3.0 mg/g extract) after the intestinal phase, thereby enhancing the compounds’ resistance and residence time in the intestinal lumen. A comparison between the addition of β‐cyclodextrin during extraction and post‐extraction revealed that, although both approaches reduce the phlorotannin loss throughout the gastrointestinal tract, the β‐cyclodextrin‐assisted extraction showed better efficacy. This indicates that strong guest‐host interactions are formed between phlorotannins and cyclodextrins during the extraction, possibly due to the increased enthalpy generated by microwave heating.

Overall, this study highlights β‐cyclodextrin‐microwave‐assisted extraction as a promising approach to enhance the stability of phlorotannins in their transit through the gastrointestinal tract, ensuring their delivery to the intestinal lumen, where they can be released and available for absorption.

## 1 Introduction

Phlorotannins stand out as an important class of polyphenols found exclusively in brown macroalgae. These compounds, built up of several phloroglucinol units, have garnered a significant interest over the recent years due to the diversity of the bioactive effects they can exert, encompassing activities such as antioxidant, anti‐inflammatory, antitumor, antidiabetic, and anti‐macular degeneration, among others (Shrestha et al. 2021).

The main route of entry of phlorotannins into our organism is through diet, which means that, when considering their actual efficacy as bioactive agents in vivo, one must account for the degree of biotransformation they may undergo while crossing the gastrointestinal (GI) tract. Recent studies have shown that the amount of phlorotannins remaining at the end of the intestinal phase of digestion is extremely low compared to their initial concentrations, with losses that can range between 60% and 80% (Catarino et al. 2022; Catarino et al. 2024; Catarino et al. 2021). Extracts of *Fucus vesiculosus* (Phaeophyceae) were reported to retain only 19% of their initial phlorotannin content, making it one of the species with the highest reported phlorotannin losses during GI digestion (Catarino et al. 2021). Such extensive degradation and transformation represent a significant limitation in terms of bioavailability, ultimately hindering the in vivo properties of phlorotannins. Strategies that can improve the integrity of these compounds during their passage through the gastrointestinal tract are therefore essential to maximize their potential health benefits.

Encapsulation of active components can offer protection against the harsh conditions of the digestive system and contribute to their release in a sustained manner in more advanced stages of the GI tract. Increased resistance to degradation by fermentation was demonstrated by encapsulating *Sargassum ilicifolium* (Phaeophyceae) phlorotannins with chitosan‐tripolyphosphate, denoting potential advantages for targeted delivery of phlorotannins to the sites of absorption (Kaushalya and Gunathilake 2022). Encapsulation of these compounds into nanofibers and other nanoscale delivered systems has also been reported, although the effects upon gastrointestinal digestion have not been thoroughly addressed (González‐Ballesteros et al. 2017; Surendhiran et al. 2019).

Formed by bacterial or enzymatic degradation of starch, cyclodextrins (CD) are natural cyclic oligosaccharides composed of D‐glucose units linked together by α‐1,4‐glycosidic bonds. Their structure resembles a truncated cone, with a hydrophobic cavity and a hydrophilic outer rim. β‐CD is composed of 7 glucose units, whereas γ‐CD forms a larger cavity composed of 8 glucose units. Guest molecules of appropriate size can interact with the hydrophobic cavity via non‐covalent forces and form adducts with improved solubility (Pinho et al. 2014). Moreover, encapsulation into CD can help stabilize guest molecules, protecting them from environmental conditions such as temperature, light, pH, and enzymatic and/or microbial activity. CD are, thus, very useful adjuvants for improving the properties of bioactive molecules, namely regarding shelf‐life, flavor‐masking, resistance to food processing, and protection against hydrolysis in the stomach (Fenyvesi et al. 2016).

They can also be used as auxiliary agents in extraction methodologies to replace the typical organic solvents required for the extraction of phenolic compounds (Shi et al. 2022). Indeed, while 70% acetone is generally considered the most efficient solvent for phlorotannin extraction (Koivikko et al. 2005), it is not compatible with food application, prompting the need for greener extraction strategies. Previous studies have addressed the use of ethanol in combination with emergent extraction techniques such as microwave‐assisted extraction (MAE) for the production of food‐safe phlorotannin‐rich extracts (Amarante et al. 2020). However, with the present study we aimed to take a step forward towards a truly green chemistry method for the extraction of phlorotannins, combining cyclodextrins with MAE as an alternative for obtaining food‐grade extracts high in phlorotannins and without the need for organic solvents. This approach may offer extraction efficiency comparable to those of conventional solvent‐based methods, while simultaneously improving the stability and delivery of phlorotannins (Pereira et al. 2021).

In this context, the aim of this work was to use CD combined with MAE for the aqueous extraction of phlorotannins from an edible species rich in phlorotannins and of great commercial relevance (*F. vesiculosus)* and to evaluate whether this approach can yield extracts endowed with controlled delivery properties, contributing to their greater stability when crossing the gastrointestinal tract.

## 2 Materials and Methods

### 2.1 Reagents

*Fucus vesiculosus* harvested in October 2021 was purchased from Algaplus Lda. The material had been previously dried and ground according to the producer's internal procedures and was stored in a sealed plastic bag at −20°C until needed. Sodium nitroprusside and sulfanilamide were ordered from Acros Organics (Hampton, NH, USA). Ascorbic acid, gallic acid, phloroglucinol, NADH, nitroblue tetrazolium, phenazine methosulfate, DMBA, α‐amylase, bile salts, pancreatin, pepsin, sodium hydrogen carbonate, and D‐glucose, were obtained from Sigma (St. Louis, MO, USA). Sodium di‐hydrogen phosphate and potassium di‐hydrogen phosphate were purchased from Panreac (Barcelona, Spain). Calcium chloride was purchased from ChemLab (Eernegem, Belgium) and 3‐phenylphenol from MERK. The standards, galacturonic acid, glucuronic acid, mannuronic acid, and guluronic acid, as well as 2‐deoxyglucose and 1‐methylimidazole, were acquired from Sigma‐Aldrich (Madrid, Spain). Sulfuric acid and sodium borohydride were purchased from Fisher Chemical (Middlesex County, MA, USA). Finally, the pharmaceutical‐grade cyclodextrins (Cavamax W8 Pharma) from Wacker‐Chemie were kindly donated by Ashland Specialty Ingredients (Düsseldorf, Germany). All organic solvents were of analytical grade, unless otherwise specified.

### 2.2 Microwave Assisted Extraction (MAE) of Phlorotannins

#### 2.2.1 Water Extracts

Ground *F. vesiculosus* (2 g, particle size <0.224 mm) was accurately weighed into a set of microwave vessels and suspended in varying volumes of water. These vessels were then placed in the microwave system (Ethos MicroSYNTH Microwave Labstation, Milestone Inc.), heated at the desired temperature for 2 min, and maintained at that temperature for a certain time period, determined according to the experimental design. Samples were maintained under atmospheric pressure and stirring throughout the extraction procedure. After microwaving, extracts were allowed to cool down to room temperature, pooled together, filtered through cotton and then through a G4 glass filter, and finally freeze‐dried.

#### 2.2.2 Ethanol Extracts

The ethanol extraction was carried out (for benchmark comparison) using the same microwave system mentioned above and following the conditions previously determined in our research group (Amarante et al. 2020).

#### 2.2.3 Experimental Design

To optimize the water extraction of phlorotannins from *F. vesiculosus* using MAE, a central composite design (CCD) was adopted for evaluation of the effects of solid/liquid ratio (g/mL, X_1_), temperature (°C, X_2_), and extraction time (min, X_3_) on the yield (%, Y_1_) and total phlorotannin content (mg/g extract, Y_2_; mg/g dry seaweed, Y_3_). Results from preliminary trials were used to select suitable values for the independent variables. Results from preliminary trials were used to select suitable values for the independent variables. A total of 16 experiments, including two replicates at the central point, were conducted in a randomized manner (Table S1). Data from this experimental design was analyzed by multiple regressions to fit the following second‐order polynomial model:

$$y=\beta_{0}+\sum_{i=1}^{k}\beta_{1}x_{1}+\sum_{i=1}^{k}\beta_{ii}x_{i}^{2}+\sum_{i\neq j=1}^{k}\beta_{ij}x_{i}x_{j}$$

where y is the predicted response; *β*_0_ is the constant coefficient; *β*_i_, *β*_ii,_ and *β*_ij_ are the linear, quadratic, and interactive coefficients of the model, respectively; and *x*_i_ and *x*_j_ are the coded independent variables. An analysis of variance (ANOVA) was carried out using STATISTICA software to determine the significance of the different variables (*p* < 0.05). To validate the accuracy of the models, experiments were carried out at the optimal conditions predicted for each response, and the obtained experimental data were compared to the values predicted by the corresponding regression model.

#### 2.2.4 Aqueous Extractions Assisted With Cyclodextrins

After determining the optimal conditions for the extraction of phlorotannins in water, the same extraction procedure was carried out, but this time with the addition of β‐CD or γ‐CD to test their effect on extraction yields and phlorotannin content. Three concentrations of each CD were added to the extraction vessels, considering the total phlorotannin content (TPhC) of the initial extracts obtained without CD, and multiplying by an excess factor of 5, 15, and 30 times the moles of phloroglucinol equivalents of the non‐encapsulated extract. Since β‐CD and γ‐CD in their native forms are poorly soluble in ethanol, this step was not carried out on ethanolic extracts.

The CD and excess factor that displayed the best overall results was further used to encapsulate the extracts a posteriori. In this case, after the MAE was completed (either aqueous or ethanolic), the solvent was evaporated, and the resultant extract was resuspended in water. Next, each extract was mixed with an equal volume of CD with a proper excess factor calculated based on the TPhC of the non‐encapsulated extract. After 3 min of stirring, the two extract‐CD mixtures were subjected to snap‐freezing with liquid nitrogen, lyophilized, and properly stored until further use.

### 2.3 Extracts Characterization

#### 2.3.1 Phlorotannins Content

The quantification of the total phlorotannin content in the samples was carried out following the 2,4‐dimethoxybenzaldehyde (DMBA) protocol previously established (Chouh et al. 2022). Briefly, 250 µL of 1% (w/v) DMBA solution (in 0.36 M HCl in glacial acetic acid) were mixed with 50 µL of each extract in a 96‐well plate. After 60 min of incubation in the dark at room temperature, the absorbance was read at 515 nm, and the TPhC was calculated using a six‐point phloroglucinol calibration curve (ranging from 7.9 to 250 µg/mL) and expressed as mg phloroglucinol equivalents (PhGE)/g of extract and PhGE/g dry seaweed (reported as mean ± standard deviation of 3 replicates). A blank was prepared using only solvent in place of standard or sample.

#### 2.3.2 Polysaccharide Content and Characterization

Polysaccharides were determined through their monomeric composition by neutral sugars released with acid hydrolysis and the analysis of their alditol acetates by gas chromatography (Coimbra et al. 1996) using a Perkin Elmer‐Clarus 400 chromatograph (PerkinElmer, Waltham, MA, USA) with a split injector (split ratio 1:60) and an FID detector, according to the method described by the same authors (Reis et al. 2023). Hydrolysis of the samples was done in triplicate, and 2‐deoxyglucose was used as an internal standard for sugar quantification. Monosaccharide results are expressed as mean molar percentages (mol %) ± standard deviation. Hexuronic acids were determined by colorimetry with 3‐phenylphenol/sulfuric acid, and values were also expressed as the mean molar percentages of three replicates ± standard deviation.

### 2.4 Solid State Studies

Infrared spectra of extracts and cyclodextrin inclusion complexes were obtained as KBr pellets in a Bruker Tensor‐27 FTIR spectroscope coupled with a Specac Golden‐Gate‐Diamond ATR. Spectra were recorded in absorbance mode in the 4000–400 cm^−1^ range with a resolution of 2 cm^−1^, co‐adding 256 scans before Fourier transformation.

Thermogravimetry (TGA) studies were performed on a Hitachi STA‐300 analyzer (Tokyo, Japan), using a heating rate of 5°C min^−1^ under an air atmosphere.

### 2.5 Gastrointestinal Digestion Simulation

Simulation of the gastrointestinal digestive system followed the reported procedure from before (Bonifacio‐Lopes et al. 2022), with slight modifications. Briefly, the process started by suspending 0.5 g of dried sample in 10 mL of distilled water, followed by a pH adjustment to 5.6–6.9 with 1 M NaHCO_3_. This solution was incubated at 37°C under constant agitation while α‐amylase at 100 U/mL was added at a rate of 0.3 mL/min (oral phase). After 2 min, the oral digest was adjusted to pH 2.0 with 1 M HCl and mixed with a simulated gastric juice consisting of 25 mg/mL pepsin and added at a ratio of 0.05 mL/mL of oral digest. This mixture was then incubated at 37°C for 1 h under constant agitation (gastric phase). The gastric digest was then adjusted to pH 6.0 using 1 M NaHCO_3_, followed by the addition of a simulated intestinal juice consisting of 2 g/L of pancreatin and 12 g/L bile salts at a ratio of 0.25 mL/mL of gastric digest. This mixture was then incubated under constant agitation for 2 h, at 37°C (intestinal phase). An aliquot of 2 mL was collected before and after each step of digestion (i.e., oral digest, gastric digest, and intestinal digest, properly stored until further use).

### 2.6 Antioxidant Activity

The radical scavenging activity of the samples was analyzed via nitric oxide radical and superoxide radical (NO^•^ and O_2_^•–^, respectively) scavenging methods, following the previously described procedures (Catarino et al. 2024). Ascorbic acid and gallic acid were the reference compounds for NO^•^ and O_2_^•–^, respectively.

## 3 Results and Discussion

### 3.1 Microwave Assisted Extraction (MAE) Optimization by Response Surface Methodology

Firstly, the aqueous extraction of phlorotannins from *F. vesiculosus* was optimized using MAE. For that, the experimental values obtained for each response (Table S1) were fitted to a quadratic polynomial model that was used to study the correlations between the independent variables and corresponding responses and ultimately predict the optimal conditions for maximizing each response factor (i.e., extraction yield), TPhC in the extract, and TPhC recovered from the dry seaweed. The models fitted to the experimental designs explain 99% of the extraction yields (R^2^ = 0.992), 89% of the TPhC in the extract (R^2^ = 0.888), and 79% of the TPhC recovered from the dry seaweed (R^2^ = 0.790). According to the regression and ANOVA analysis (Table 1), the extraction yield was significantly affected by the solid/liquid ratio, temperature (both linear and quadratic factors), and the interaction between these two independent variables (*p* < 0.05), while the extract's TPhC and dry seaweed's TPhC were only significantly affected by the temperature quadratic factor (*p* < 0.05).

**TABLE 1:** Regression coefficients and analysis of variance of uncoded units for mass yield and phlorotannins content, expressed as phloroglucinol equivalents, per dry weight of extract, and per dry weight of algae.

	Yield (%)		mg PhGE/g DW extract		mg PhGE/g DW algae	
	estimate	*p*‐value	estimate	*p*‐value	estimate	*p*‐value
β_0_	53.32	**0.0002**	2.45	0.374	0.92	0.235
β_1_	−1346.54	**0.033**	−60.42	0.764	−22.34	0.687
β_11_	11711.79	0.382	−1345.45	0.792	−353.27	0.801
β_2_	−0.21	**0.020**	0.05	0.110	0.01	0.105
β_22_	0.001	**0.002**	−0.0004	**0.014**	−0.0001	**0.023**
β_3_	−0.37	0.699	0.19	0.617	−0.07	0.526
β_33_	−0.002	0.977	−0.01	0.531	0.01	0.230
β_12_	6.51	**0.005**	0.61	0.357	0.26	0.165
β_13_	−10.91	0.638	5.40	0.556	−1.08	0.665
β_23_	0.01	0.059	−0.0008	0.578	−0.0003	0.469
R^2^	0.992		0.888		0.790	

*Note*: β0 is the constant coefficient; β_1_, β_2,_ and β_3_ are the linear coefficients; β_11_, β_22_ and β_33_ are the quadratic coefficients; β_12_, β_13,_ and β_23_ are the interactive coefficients; Bold value indicates *p* < 0.05; PhGE‐ phloroglucinol equivalents; DW‐dry weight.

The optimal conditions for maximizing each response were estimated using the regression equation fitted to the experimental data obtained, and the results can be consulted in Table S2.

The combination of factors was selected so that the best combined outcome for the three responses was achieved. Accordingly, the optimal conditions determined consisted of a solid/liquid ratio of 1:100, a temperature of 130°C, and an extraction time of 3 min, which afforded an extraction yield of 47% ± 1%, a TPhC in the extract of 3.0 ± 0.5 mg PhGE/g and a TPhC of 1.4 ± 0.5 mg PhGE/g, extracted from the dry seaweed (Table 2). This means that there is a good conformity observed between experimental and predicted values (48%, 2.8 mg PhGE/g extract, and 1.0 mg PhGE/g dry seaweed, respectively), thus validating the good predictive capacity of these models.

**TABLE 2:** Yield of extraction, phlorotannins and carbohydrates content of optimized MAE‐assisted water and ethanol extracts (MAE‐H_2_O and MAE‐EtOH, respectively).

		Phlorotannins			Carbohydrates					
Extract	Yield (%)	mg PhGE/g DW extract	mg PhGE/g DW algae	Total (%)	Fuc	Xyl	Man (Molar %)	Gal	Glc	HexA
MAE‐H_2_O	47 ± 1	3.0 ± 0.5	1.4 ± 0.5	28 ± 3	22 ± 1	2 ± 0.1	34 ± 2	4 ± 0.1	8 ± 0.4	29 ± 1
MAE‐EtOH	35 ± 1	4.4 ± 0.6	1.6 ± 0.3	24 ± 3	6 ± 0.2	—‐	66 ± 0.1	5 ± 0.4	14 ± 1	8 ± 1

**Abbreviations**: PhGE‐ phloroglucinol equivalents; DW‐dry weight; Fuc‐fucose; Xyl‐xylose; Man‐mannose; Gal‐galactose; Glc‐glucose; HexA‐hexuronic acids; results are expressed in mean ± standard deviation.

### 3.2 Characterization of Food Grade (Water and Ethanol) *Fucus vesiculosus* Extracts

#### 3.2.1 Phlorotannins and Polysaccharides Content

The latest proposed method using a solvent accepted in food applications, the MAE‐EtOH optimized by Amarante et al. (2020), resulted in an extract with a yield of 35% and a TPhC of 4.4 mg PhGE/g extract. In the present study, the optimization of an identical process using only water led to a clear improvement in terms of overall extraction efficiency, with yields 12% higher than the ethanolic extraction (Table 2). Although the MAE‐EtOH showed a slightly higher specificity for phlorotannins as reflected in its higher TPhC, the fact that both extraction methods were able to extract similar amounts of phlorotannins from the seaweed matrix (1.4 ± 0.5 and 1.6 ± 0.3 mg PhGE/g dry seaweed, respectively) demonstrates that the MAE‐H_2_O extraction is as effective as MAE‐EtOH in obtaining these compounds from seaweed.

The lower TPhC observed for MAE‐EtOH in this study, compared to Amarante et al. (2020), may be due to seasonal variation, as the seaweed was harvested in autumn (October 2021) rather than summer, when phlorotannin levels are typically higher in brown macroalgae (Connan et al. 2004; Parys et al. 2009; Stiger et al. 2004).

The content of carbohydrates of the extracts was slightly higher in MAE‐H_2_O compared to MAE‐EtOH, with the former containing mainly Fuc (22 mol %), Man (34 mol %), and HexA (29 mol %), most likely corresponding to guluronic acid from alginic acid (Table 2). This composition was expected and is coherent with the composition of brown seaweed polysaccharides, reflecting the presence of fucoidans, alginic acid, and mannitol, which are highly soluble in hot water (Xie et al. 2024).

In turn, the polysaccharide profile of MAE‐EtOH was dominated by Man (66 mol %), mostly from mannitol, because ethanol hampers the solubilization of the polysaccharides mentioned above (Guo et al. 2020; Zayed et al. 2016). The lower ability of ethanol to dissolve polysaccharides suggests the lower extraction yields observed in the MAE‐EtOH in comparison with the MAE‐H_2_O.

#### 3.2.2 Antiradical Activity

**FIGURE 1:**
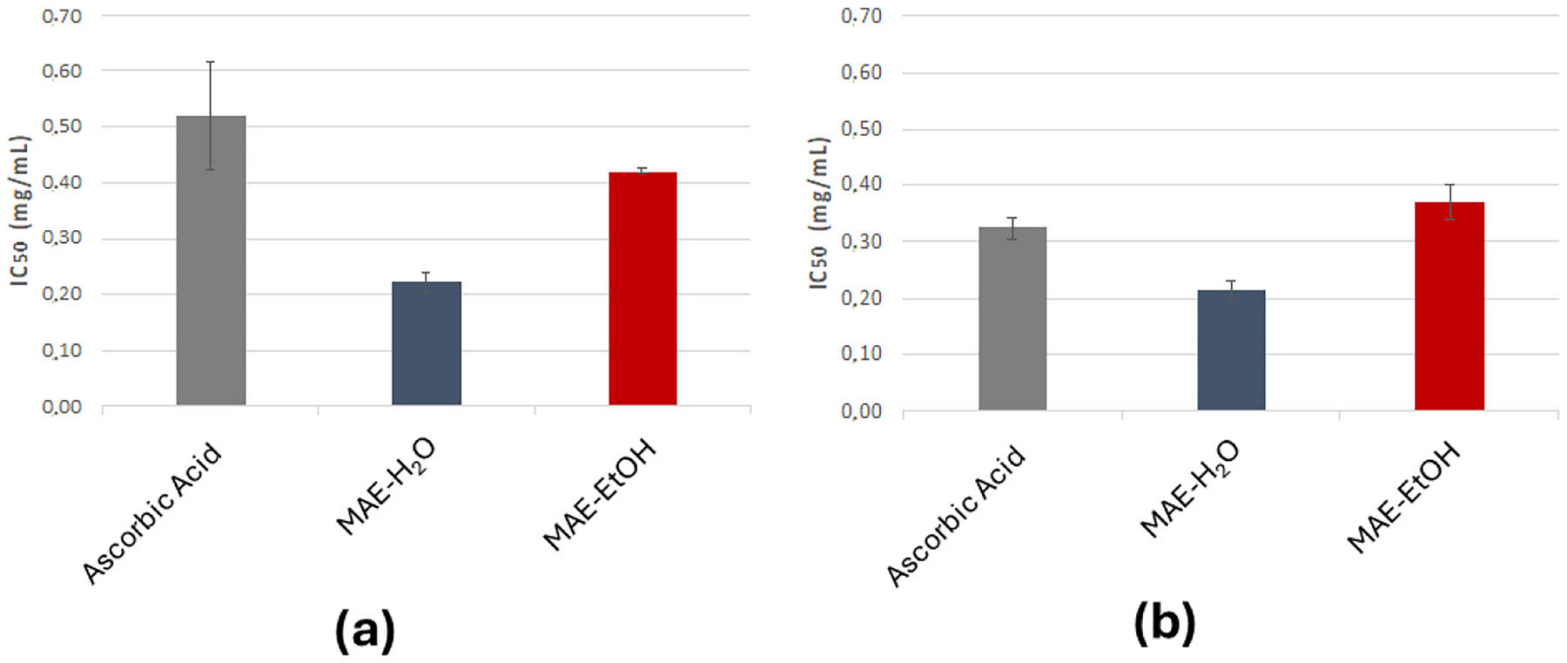
Antioxidant activities of *F. vesiculosus* MAE‐H_2_O and MAE‐EtOH measured via (a) NO^•^ scavenging activity and (b) O_2_^•–^ scavenging activity. Data is represented in mean ± standard deviation and the results are expressed in concentration (in mg/mL) necessary to inhibit 50% of the radicals (IC_50_).

Since MAE‐H_2_O was nearly as effective as MAE‐EtOH in extracting phlorotannins from *F. vesiculosus* (despite its lower specificity), the next step was to evaluate whether both extracts would exhibit comparable antioxidant activity. For that, both extracts were screened for their capacity to scavenge the NO^•^ and O_2_^•–^, corresponding to two radicals of great biological importance, as they are an integral part of several human physiological mechanisms, including inflammation and oxidative stress. As depicted in Figure 1, both extracts displayed excellent scavenging activity against the tested radicals, with IC_50_ values equivalent to or even lower than those of ascorbic acid, used as a reference compound. A slightly stronger scavenging potency was observed in the NO^•^ assay compared to the O_2_^•−^, particularly considering the effects of the standard compound. Similar results have been reported using conventional 70% acetone extract or fractions of an 80% ethanolic extract from the same species, which also displayed NO^•^ scavenging activities comparable to or exceeding that of ascorbic acid (Catarino et al. 2020; Wang et al. 2012).

High activity often correlates with the TPhC of the extracts, with higher TPhC values indicating greater potential for radical scavenging and reductive capacity, and consequently higher antioxidant activities. However, the values observed suggest that the relationship between TPhC and antioxidant activity might depend on other factors such as structural features of the compounds extracted and the polysaccharide profile. Indeed, even though the TPhC levels of MAE‐H_2_O are lower, it exhibited a superior scavenging effect (NO^•^ 0.22 mg/mL; O_2_^•−^ 0.21 mg/mL) compared to MAE‐EtOH (NO^•^ 0.42 mg/mL; O_2_^•−^ 0.37 mg/mL). These results are likely related to the differences in the structures of the extracted phlorotannin, as each solvent can influence the phlorotannin profile and affect the extract's antioxidant properties. The DMBA assay herein used is not, however, specific enough to comprehensively evaluate and elaborate accurate conclusions over these structural‐activity nuances. To fully elucidate the relation between activity and specific molecular weight features, more advanced techniques such as HPLC‐MS or nuclear magnetic resonance would be necessary.

Previous studies have shown that organic solvents like ethanol or acetone preferentially extract high molecular weight phlorotannins, while water, in turn, tends to favor the extraction of medium molecular weight oligomers (Koivikko et al. 2005). These oligomeric forms usually possess optimal spatial configurations for radical‐trapping and display greater efficacy compared to larger polymeric structures. In fact, results reported by Gisbert et al. (Gisbert et al. 2022) demonstrated that phlorotannin fractions from *Ascophyllum nodosum* with polymerization degrees between 4 to 17 units of phloroglucinol displayed the highest antioxidant activity.

Furthermore, as depicted in Table 2, the extraction solvent significantly impacted the co‐extraction of other bioactive compounds, specifically polysaccharides. MAE‐H_2_O presented considerably higher levels of fucose and hexuronic acids compared to MAE‐EtOH, indicating higher abundance in fucoidans and alginates. Indeed, sulfated polysaccharides (fucoidans) and alginates have been extensively documented in the literature for their intrinsic antioxidant properties, including radical scavenging and metal chelating activities (El‐Sheekh et al. 2024; Wang et al. 2008). Therefore, higher levels of these compounds in the aqueous extract likely contribute synergistically to the overall antioxidant effect observed.

Consequently, even if MAE‐H_2_O has slightly lower TPhC compared to MAE‐EtOH, the advantageous structural profile of the phlorotannins combined with the co‐extraction of antioxidant polysaccharides reinforces that water can be used as a viable, sustainable, and food‐grade alternative to organic solvents to obtain extracts that maintain identical or even improved bioactivity.

### 3.3 MAE of Phlorotannins With Water and Addition of Cyclodextrins

To investigate the effect of the addition of increasing concentrations of cyclodextrin during the MAE and to evaluate the effects on extraction yield and TPhC, inclusion complexes were prepared. Inclusion of pure guests typically employs well defined stoichiometries, often in equimolar quantities when the guest has an adequate size to fit into the cavity of one CD. However, when the objective is to extract compounds from natural sources, it is impossible to use the same conditions due to the inability to accurately estimate the size and quantity of each bioactive compound that will be present in the extract, as well as predict all the other co‐extracted compounds such as pigments, terpenes, proteins, and other small molecules that can interact with CDs and influence the inclusion efficacy. The amount of CD added to the extraction mixture was determined based on the amount, in moles, of phloroglucinol equivalents quantified in the non‐encapsulated extract multiplied by an excess factor of 5, 15 and 30 times. Because CDs are poorly soluble in ethanol and the purpose is to reduce the use of organic solvents, CD‐MAE studies were conducted exclusively with water as a solvent.

Results presented in Table 3 show that both β‐CD × 5 and γ‐CD × 5 had no significant impact on extraction yields or TPhC compared to the MAE‐H_2_O extract alone. However, as the concentrations of CD were further raised, a corresponding rise in extraction yields was observed. Additionally, it is worth noting that both β‐ and γ‐CD afforded very similar yield values at the same concentrations.

**TABLE 3:** Extraction yields and total phlorotannin content of the *F. vesiculosus* aqueous extracts obtained via different CD‐MAE conditions.

Cyclodextrin	Yield (%)	mg PhG/ g DW extract	mg PhG/ g DW algae
γ‐CD × 5	47 ± 2	2.8 ± 0.2	1.3 ± 0.1
γ‐CD × 15	49 ± 2	2.0 ± 0.2	1.0 ± 0.1
γ‐CD × 30	55 ± 1	1.4 ± 0.2	0.8 ± 0.1
β‐CD × 5	47 ± 3	2.8 ± 0.3	1.3 ± 0.1
β‐CD × 15	51 ± 1	2.4 ± 0.3	1.2 ± 0.1
β‐CD × 30	54 ± 2	1.7 ± 0.2	0.7 ± 0.1

**Abbreviations**: CD—cyclodextrin; DW—dry weight; PhG—phloroglucinol equiv.; results are expressed in mean ± standard deviation.

Interestingly, an inverse proportionality was noted between the CD concentration and TPhC of the extracts (i.e., the higher the CD concentration, the lower the TPhC). Similar outcomes have been previously described in aqueous extracts of grape pomace in which the addition of α‐CD, β‐CD, or γ‐CD all displayed lower total phenolic content than the conventional extract obtained with EtOH 80% (Ratnasooriya and Rupasinghe 2012). In this case it should be noted that guests with high affinity to the CD cavity can establish stronger interactions, which can hamper their release to the reaction medium and hinder their quantification, thus explaining the lower TPhC values herein observed, despite the higher extraction yields. It is also possible that CDs are facilitating the extraction of other non‐phlorotannin compounds and that way reducing extraction selectivity towards phlorotannins, consequently reducing their concentration in the extract.

#### 3.3.1 Solid‐State Analysis by Infrared Spectroscopy and Thermogravimetry

Solid/dried samples of the extracts obtained in the presence of β‐CD or γ‐CD were analyzed by infrared spectroscopy (FT‐IR) and thermogravimetry (TGA).

**FIGURE 2:**
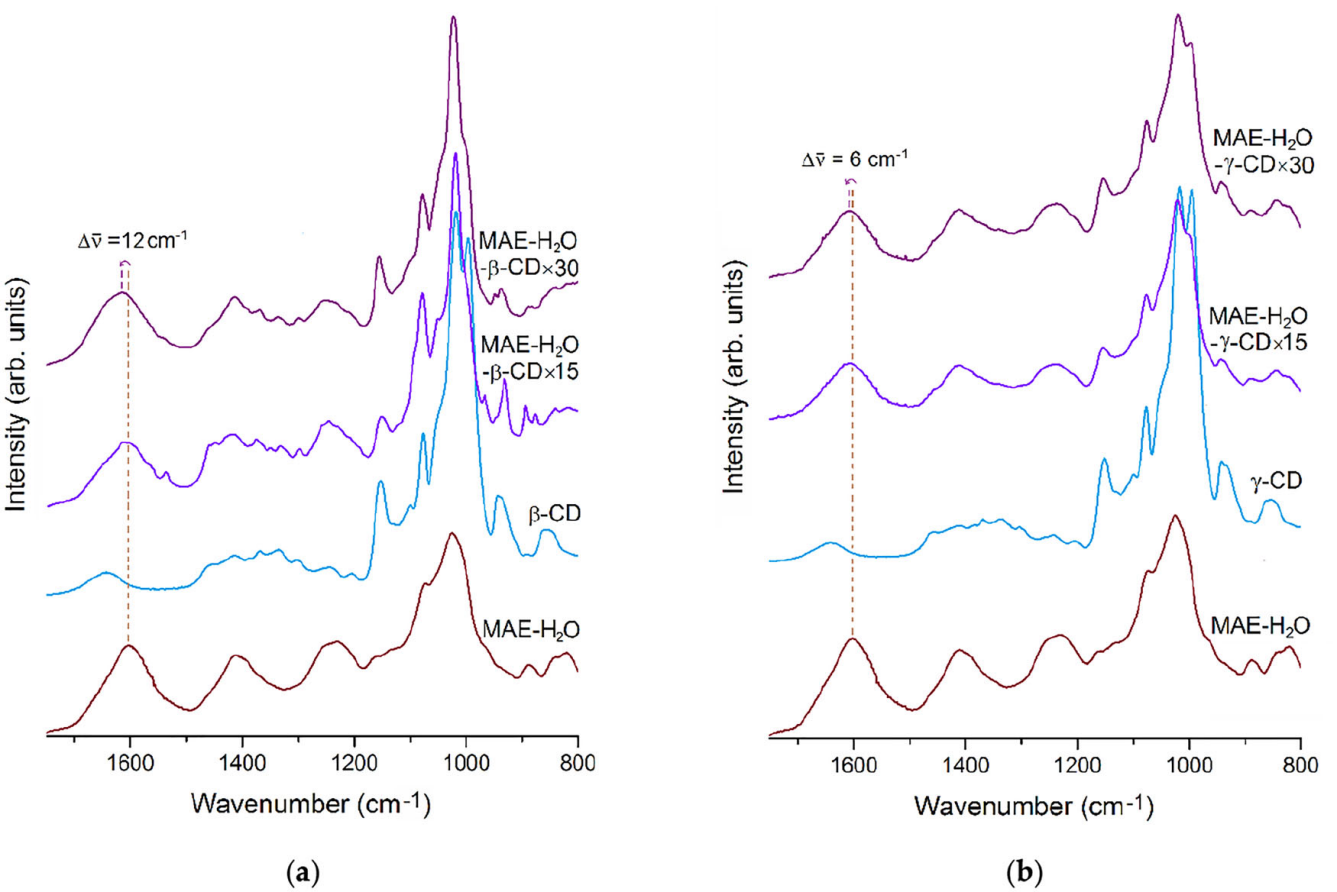
Infrared spectra, in the selected region of 1750 – 800 cm^−1^, of (a) the pure aqueous extract of *Fucus vesiculosus* (MAE‐H_2_O), β‐cyclodextrin, and dry samples of MAE‐H_2_O‐β‐CD×15 and MAE‐H_2_O‐β‐CD×30; (b) MAE‐H_2_O, γ‐cyclodextrin, and dry samples of MAE‐H_2_O‐γ‐CD×15 and MAE‐H_2_O‐γ‐CD×30.

The FT‐IR spectra of extracts prepared in the presence of CD (Figure 2) show the presence of a broad band in the region between 1700 and 1500 cm^−1^, ascribed to the phenolic components of the extract. Note that, in the pure aqueous extract (MAE‐H_2_O sample), this band peaked at 1603 cm^−1^, while in the samples MAE‐H_2_O‐γ‐CD × 15 and MAE‐H_2_O‐γ‐CD × 30, it peaked at 1609 cm^−1^. This is likely the result of the inclusion of some of the phenolic components into the cavity of γ‐CD. In samples MAE‐H_2_O‐β‐CD × 15 and MAE‐H_2_O‐β‐CD × 30, the band ascribed to the phenolic components presented an even stronger blueshift, peaking at 1615 cm^−1^; this can be attributed to inclusion into the narrower cavity of β‐CD, which causes a higher increase in the vibrational energy associated with the stretching modes of the phenolic rings. Noteworthy, all the samples containing extracts exhibit evidence of the presence of polysaccharides originating from the seaweed extract, perceived as the presence of a very sharp peak at 1027 cm^−1^, dominant in this spectral region.

**FIGURE 3:**
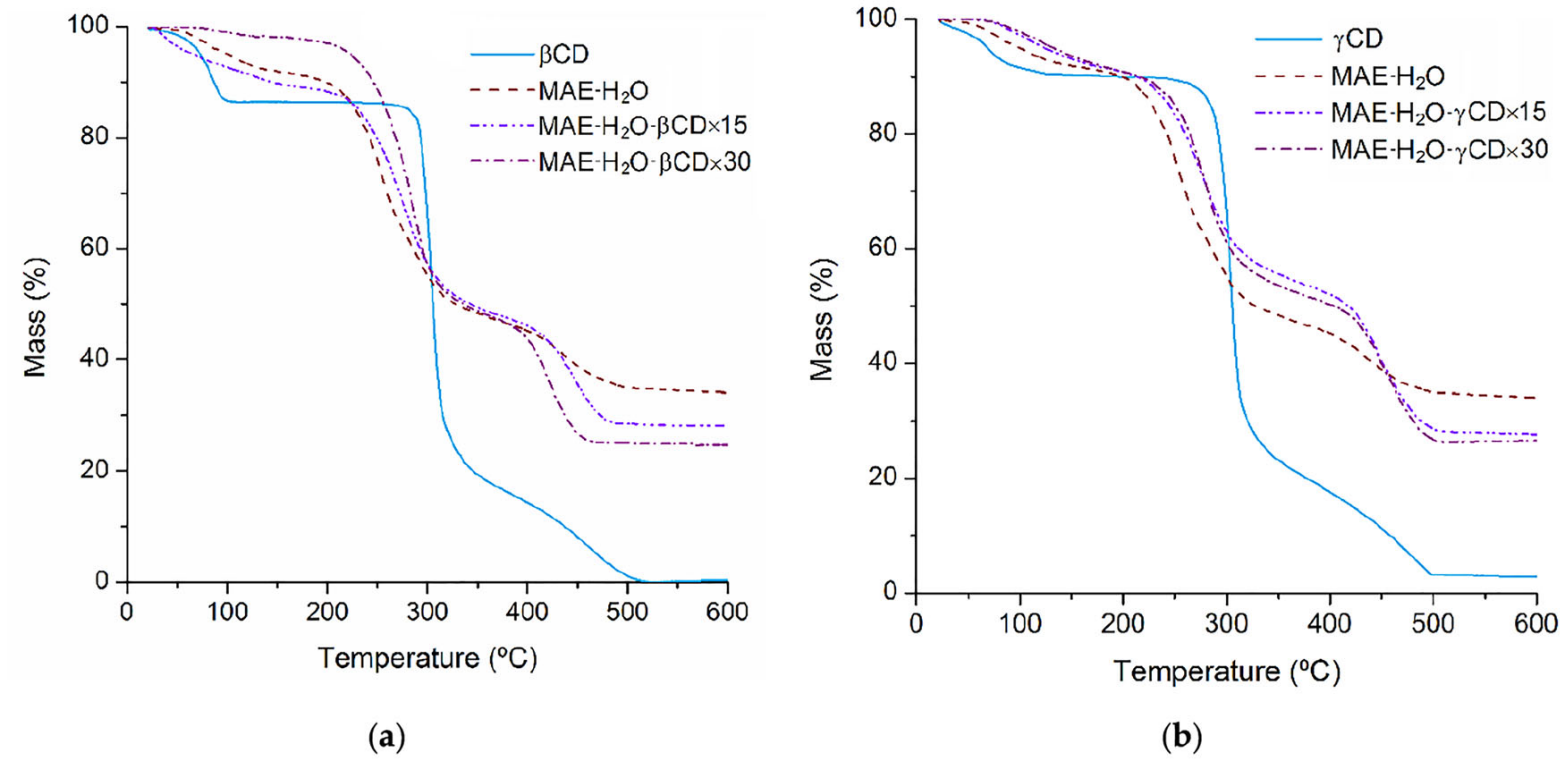
Thermogravimetric traces of (a) the pure aqueous extract (MAE‐H_2_O), β‐cyclodextrin, and dry samples of MAE‐H_2_O‐β‐CD × 15 and MAE‐H_2_O‐β‐CD × 30; and (b) the pure aqueous extract (MAE‐H_2_O), γ‐cyclodextrin, and dry samples of MAE‐H_2_O‐γ‐CD × 15 and MAE‐H_2_O‐γ‐CD × 30.

The TGA data of the same set of samples is presented in Figure 3. TGA traces of the samples are characterized by an early mass loss in the interval between ambient temperature and *c.a*. 115°C, ascribed to the loss of hydration water molecules. Note that the step is more pronounced for β‐CD, which typically features 10 hydration waters per molecule, with γ‐CD having a slightly smaller dehydration step, associated with *c.a*. 6–7 water molecules. The extracts, whether pure MAE‐H_2_O or those prepared in the presence of CDs, exhibit very small dehydration steps. This difference can be attributed to the higher hydrophobicity of these samples that is brought about by the presence of the phenolic compounds and renders them less prone to accumulate water molecules. The residual char of the aqueous extract is around 34%–35% of its total mass, with those of the cyclodextrin‐assisted extracts presenting lower values, around 28% for MAE‐H_2_O‐β‐CD × 15 and 24% for MAE‐H_2_O‐β‐CD × 30, which is in agreement with the increasing percentage of β‐CD in these samples and with the fact that this CD fully decomposes at around 530°C (leaving no char residue). Similarly, char mass for the extracts obtained with γ‐CD (×15 and ×30) also presents a reduction in percentage, to values of 28% and 26%, respectively, of the total mass.

Overall, the smaller dehydration steps in the thermograms of the extracts assisted with CDs, particularly the notorious sample MAE‐H_2_O‐β‐CD × 30, together with the shifts observed in the FT‐IR spectra, are indicative of interaction between the CDs and phlorotannins. Different types of interaction are likely to be occurring; molecules of adequate size to fit the host cavity can form inclusion compounds, while hydrogen bonding and other non‐inclusion interactions may be occurring with phlorotannins of larger dimensions. Taken as a whole, it is expected that these interactions contribute to increased stability of the phlorotannins in the cyclodextrin‐containing samples, it also being possible that they will contribute to preventing phlorotannin degradation during GI digestion.

#### 3.3.2 Gastrointestinal Digestion Simulation

It is well‐known that, during gastrointestinal digestion, polyphenols face several extreme conditions, such as pH variations, exposure to enzymatic activity, complexation with proteins, and microbial metabolization, among others. These factors can significantly affect polyphenol structure, biological activity, and bioavailability, namely in what concerns their absorption into the bloodstream (Sanchez‐Velazquez et al. 2021). In turn, cyclodextrins can greatly improve the stability of compounds while they are transiting over the gastrointestinal tract, protecting them from the gut's harsh conditions and facilitating their delivery to the target site in what is deemed sustained release (Hu et al. 2021). To study the effect of the presence of CD in the stability of the phenolic content, the different aqueous extracts, that is, pure MAE‐H_2_O and those obtained in the presence of CD, were subjected to an in vitro gastrointestinal digestion protocol and analyzed regarding their TPhC after each gut compartment.

**FIGURE 4:**
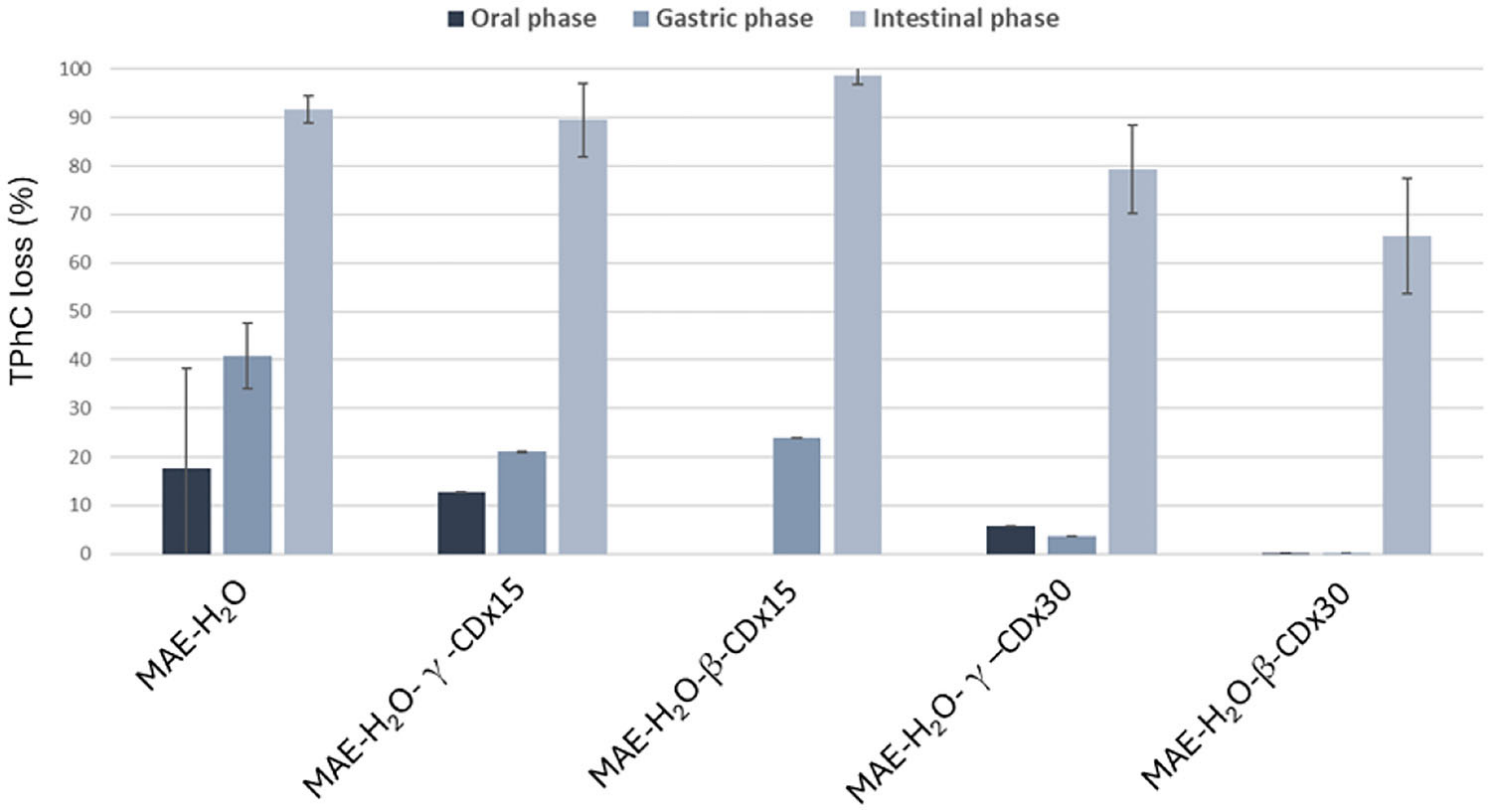
Loss in percentage (%) of the total phlorotannin content (TPhC) on the MAE water extract (MAE‐H_2_O) and in extracts obtained in the presence of cyclodextrins (γ and β ×15 and ×30) after oral, gastric and intestinal phases. Data represent the mean ± standard deviation and the results are expressed in percentage of TPhC loss.

After each step of the gastrointestinal digestion, an increasing loss of TPhC was noticed on MAE‐H_2_O (Figure 4), ending up at 0.236 mg PhGE/g DW extract by the end of the intestinal phase. These observations follow an identical pattern when comparing with previous studies on the impact of digestion on phlorotannins extracted from different species of brown seaweed (Catarino et al. 2022; Catarino et al. 2024; Catarino et al. 2021). Notably, loss in TPhC was lower in the extracts obtained using CD. Specifically, the losses in TPhC in the stomach compartment for the sample MAE‐H_2_O_‐_γ‐CD×15 were only 21% ± 0.1% comparing with the 41% ± 7% observed for the MAE‐H_2_O extract at the same stage of digestion, representing a reduction of almost 50% in the amount of phlorotannins lost. These results can be attributed to the sustained release effect of γ‐CD, which protects phlorotannins from degradation during the first stages of the gastrointestinal digestion, releasing them mostly during the intestinal phase. Similarly, in the extract MAE‐H_2_O‐β‐CD × 15 there was also evidence of the protective role of β‐CD, with the added benefit that in the oral phase there was no loss in TPhC whatsoever. This is a result of the strong interaction of β‐CD with the polyphenols resulting from the tight fit into the narrow host cavity, as previously mentioned in the description of the FTIR results. Moreover, it indicates that the sustained release in the case of β‐CD occurs more gradually compared with that of γ‐CD, allowing phlorotannins to pass through the oral digestion without suffering any decomposition. As oral digestion only takes a brief moment and longer residence times, like in the gastric or intestinal phases, it might be necessary to achieve a more quantitative release of the encapsulated phlorotannins and coextracted bioactive compounds.

The increase in CD concentration to an excess factor of × 30 further improved the protection of TPhC from degradation in the in vitro simulated digestion assay. Not only was TPhC loss during the oral and gastric phases significantly low (to values very close to 0%), but there was also a noticeable protective effect on the intestinal phase (approximately 20%), which did not occur for the extracts with CD × 15. Once again, the extract with β‐CD (MAE‐H_2_O‐β‐CD × 30) performed slightly better than the equivalent one with γ‐CD. Apart from preventing the loss of TPhC in the oral and gastric phases to negligible values, it also reduced the TPhC loss in the intestinal phase to below 70%, thus affording a substantial difference in the sustained‐release properties. Such observations suggest that, in these conditions, the presence of cyclodextrins protects phlorotannins from oral and gastric conditions and consequently increases their resistance and residence time in the intestinal lumen. The amount of phlorotannins available for absorption is, therefore, increased.

While there are no previous studies on how CD can affect the stability of phlorotannins along the gastrointestinal tract, the results herein reported are concordant and supported by relevant studies carried out with other polyphenols. Indeed, Fernandes et al. (2018) showed that the incorporation of β‐CD in a blackberry puree greatly reduced its anthocyanin degradation index at the end of the gastrointestinal digestion, while an in vivo experiment demonstrated that the encapsulation of piceatannol in α‐CD nearly doubled the rate of recovery of the compounds in gastric juice and quadrupled it in the lower small intestine (Inagaki et al. 2016). Concerning tannins, a study on a combination of green tea catechins, ascorbic acid, and xylitol that was encapsulated into γ‐CD showed an enhancement of the compounds’ digestive stability of 65%, and a 9% increase in the absorption rates compared with the non‐encapsulated formula (Son et al. 2016). In turn, the protective effects of CDs on phlorotannins remain largely unexplored, although a recent study showed that γ‐CD inclusion of phloroglucinol (the phlorotannin basic unit) enhanced its stability and intestinal transport while preserving the compound's bioactive properties, particularly its antioxidant activity (Catarino et al. 2022).

Notably, while phlorotannins are not fully absorbed in the intestinal lumen, CDs may help protect these compounds from intestinal degradation, potentially allowing higher amounts to reach the large intestine and to interact with the gut microbiota, modulating their growth and/or activity, or suffering microbial metabolization and conversion into different bioactive metabolites capable of entering the bloodstream and exerting health‐promoting effects.

#### 3.3.3 Effect of Cyclodextrin on the Digestion of Bioactive Extracts

To maximize phlorotannin stability and resistance to digestion, the effect of adding CD was evaluated during or after the microwave‐assisted extraction. The β‐CD with the excess factor of ×30 was the selected condition for these studies because it displayed the best protective effects on the host during the previous experiments on gastrointestinal digestion. The ethanolic extract was also studied for comparison, but only after microwave‐assisted extraction due to the fact that β‐CD is insoluble in ethanol, which makes it impossible to test the addition of β‐CD during the ethanolic microwave‐assisted extraction process.

**FIGURE 5:**
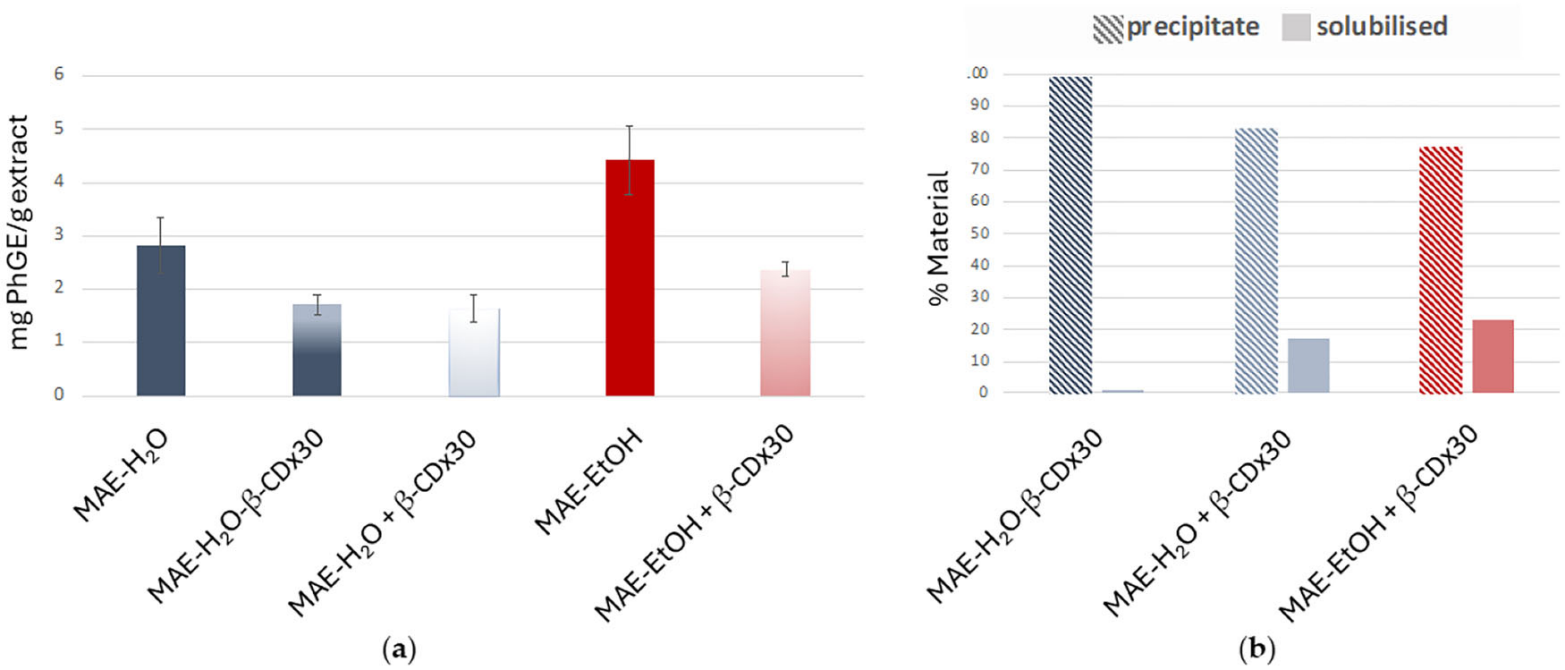
(a) Total mg of phloroglucinol equivalents (PhGE)/g extract and (b) % material solubilized and precipitate after ethanol precipitation. Data represents the mean ± standard deviation.

As shown in Figure 5a, the TPhC in the MAE‐H_2_O‐β‐CD × 30 extract was lower compared to that of the MAE‐H_2_O extract, in good agreement with the previous observations made in Section 3.3. Interestingly, a similar trend was observed when encapsulation was performed after extraction, as the addition of β‐CD × 30 to MAE‐H_2_O (MAE‐H_2_O **+** β‐CD × 30) extract also resulted in lower TPhC values. In fact, the TPhC determined for this extract closely matched the values measured for the MAE‐H_2_O‐β‐CD × 30 extract. This phenomenon was also observed for the ethanolic extract since the MAE‐EtOH extract followed by the addition of β‐CD × 30 (MAE‐EtOH **+** β‐CD × 30) similarly led to lower TPhC levels.

These observations support the hypothesis that the phlorotannins in these extracts might establish strong interactions with the hydrophobic cavity of the β‐CD and consequently hinder their quantification through the DMBA method. Indeed, previous studies from our research group demonstrated that phloroglucinol (i.e., the base unit of phlorotannins) displayed a strong affinity with γ‐CD, allowing them to form an in situ adduct when both phloroglucinol and γ‐CD were physically mixed together (1:1) (Catarino et al. 2022). Therefore, it is likely that other phlorotannins may also display similar guest‐host affinities that could explain these results.

Encapsulation of bioactive molecules into cyclodextrins induces alterations in their physicochemical characteristics, which can affect their chemical reactivity, stability, and solubility (Inagaki et al. 2016). In this context, all the CD‐containing extracts were mixed with ethanol to precipitate the β‐CD and evaluate the percentage of soluble and insoluble material in the solution, and in turn the strength of interactions. According to Figure 5b, MAE‐H_2_O‐β‐CD × 30 exhibited low solubility in ethanol, in contrast to the other two extracts where β‐CD was added post‐extraction. Among these latter extracts, MAE‐EtOH **+** β‐CD × 30 showed a slightly higher percentage of soluble material compared to the MAE‐H_2_O‐β‐CD × 30. This finding is consistent with the fact that the extracts obtained using ethanol contain compounds that are more soluble in this solvent and, therefore, are more easily released from the complex with β‐CD upon re‐suspension in ethanol. The most intriguing observation, however, was that the addition of β‐CD during and after microwave‐assisted extraction afforded notably distinct results in terms of solubility in ethanol. This suggests that the presence of β‐CD during the extraction can promote stronger interactions with the extracted compounds that may result from the increased enthalpy generated by the microwave conditions and high temperatures applied during the extraction process (Inagaki et al. 2016). Indeed, previous studies have reported that the use of microwave irradiation can significantly improve encapsulation efficiency, namely for chrysin and thymol, as well as in more complex matrixes such as essential oils (Das et al. 2020; Hernandez‐Sanchez et al. 2017; Rodríguez‐López et al. 2019).

Heating is known to increase the solubility of compounds during the extraction process, as well as to improve the solubility of β‐CD, the native CD with the lowest aqueous solubility at room temperature (Loftsson and Brewster 2012). Combining these two effects leads to higher complexation efficiencies. In addition, the sub‐critical state of water can create an environment where less polar compounds have higher solubility, thus enhancing the mass transfer from the seaweed matrix to the medium and facilitating their access to the hydrophobic cavity of the CD (Munir et al. 2018).

**FIGURE 6:**
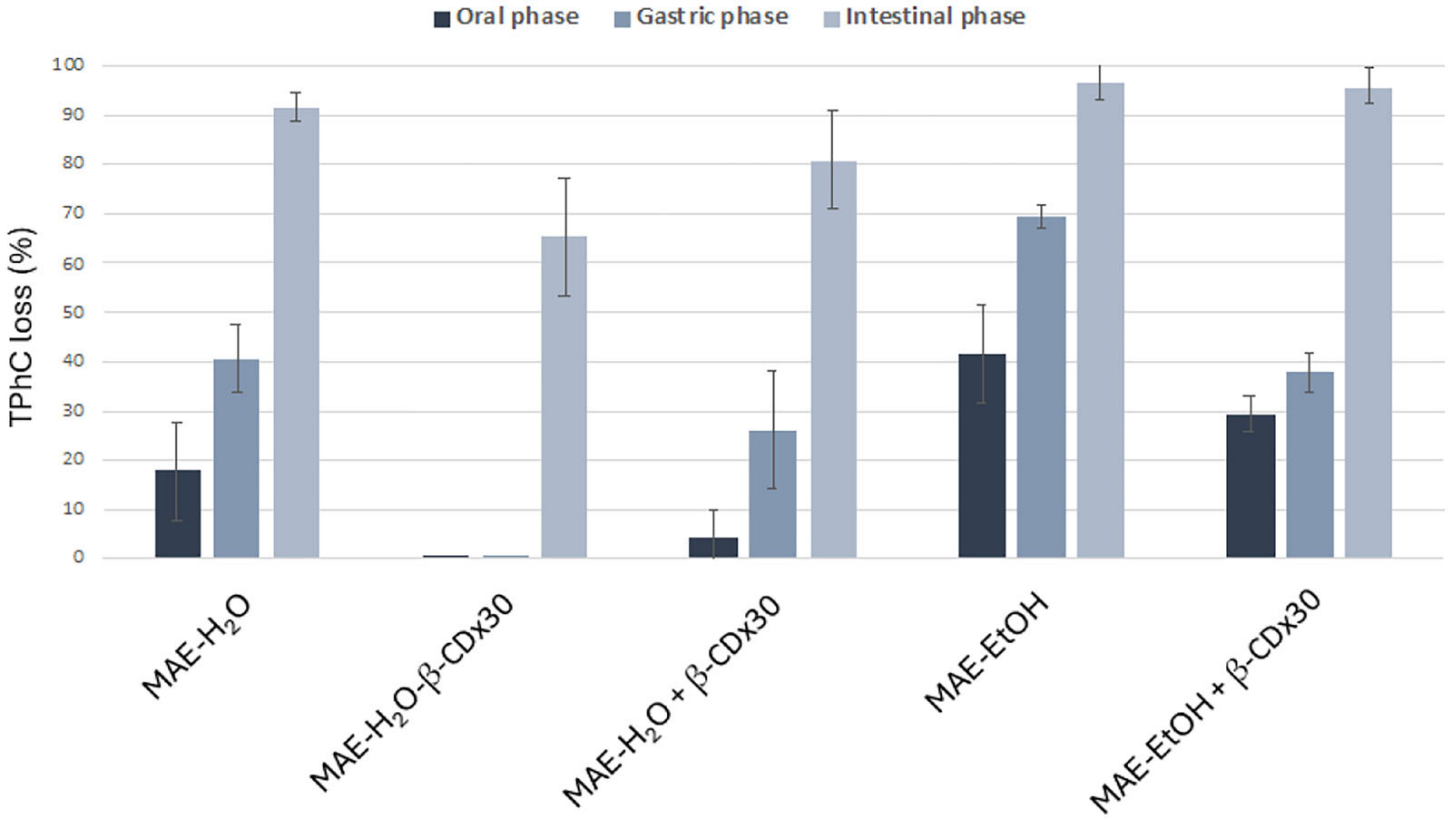
Loss in percentage (%) of the total phlorotannin's content (TPhC) on the MAE aqueous and ethanol extracts (MAE‐H_2_O and MAE‐EtOH), and their β‐CD x30 versions after oral, gastric and intestinal phases.

Once again, the Infogest protocol was applied to the samples to evaluate TPhC loss over the different stages of the GI digestion. As shown in Figure 6, there was a clear difference between the aqueous and ethanol extracts. In general, ethanol extracts revealed a significantly higher rate of phlorotannin loss throughout the gastrointestinal tract, reaching up to nearly 100% loss at the intestinal level. Nevertheless, MAE‐EtOH **+** β‐CD × 30 contributed to slowing down the TPhC loss during the oral and gastric phases, allowing a higher percentage of phlorotannins to be released in the intestinal phase, although, at the end of the intestinal digestion, the TPhC loss was nearly complete. Interestingly, the levels of TPhC loss for MAE‐EtOH **+** β‐CD × 30 followed a similar pattern as that of MAE‐H_2_O, suggesting that the composition of the latter (for instance, the presence of polysaccharides) contributes with the protective and stabilizing effects towards the phlorotannins present in it. Therefore, using CD to improve the stability of phlorotannins in MAE‐EtOH may be unnecessary, because a simple change of the solvent to an aqueous one affords better results. Moreover, it reduces costs, and it is environmentally advantageous.

For the water‐extracted samples, in turn, the addition of β‐CD × 30, either during or after the MAE, significantly contributed to reducing the loss of TPhC across the gastrointestinal tract, although to different extents. Indeed, MAE‐H_2_O **+** β‐CD × 30 showed a significant decrease in the loss of TPhC at all stages of the GI digestion, ending the intestinal stage with still 20% of the TPhC remaining against the 8% observed in the pure MAE‐H_2_O extract. However, as observed above, better results were achieved with the extract MAE‐H_2_O‐β‐CD × 30, as it completely preserved TPhC at the oral and gastric phases and decreased the loss of TPhC at the intestinal level from 81% to 66%. Again, this could be explained by the formation of inclusion complexes with a tighter fit and, thus, stronger affinity in the sample MAE‐H_2_O‐β‐CD × 30, as well as the protective effect of having a larger excess of β‐CD in this sample (available for multiple intermolecular non‐inclusion interactions). This provided higher protection against degradation during the passage through the gastrointestinal tract. Moreover, such results are coherent with the solubility studies, in which MAE‐H_2_O‐β‐CD × 30 was the extract that simultaneously demonstrated lower material solubility in EtOH and fewer losses in TPhC. The fact that the other two extracts (i.e., MAE‐EtOH **+** β‐CD × 30 and MAE‐H_2_O **+** β‐CD × 30) exhibited higher solubility of the extract material can represent higher exposure to the GI digestion conditions and partially explain the observed losses in TPhC.

## 4 Conclusion

This study demonstrates the utility of a cyclodextrin‐assisted method in the aqueous microwave‐assisted extraction as an effective strategy to enhance the extraction yield and the stability of phlorotannins from *Fucus vesiculosus*, measured as the loss in total phenolic content in an in vitro method simulating the passage through the gastrointestinal tract. While higher cyclodextrin concentrations improved extraction yields, a trade‐off was observed with total phlorotannin content, although this was most likely attributed to strong phlorotannin‐CD interactions that led to underestimated values.

β‐CD exhibited significant protective effects against phlorotannin degradation, especially in the oral and gastric phases, with a notable reduction in the losses occurring at the intestinal phase. Comparing the addition of β‐CD during extraction (MAE‐H_2_O‐β‐CD × 30) and the addition of β‐CD post extraction (MAE‐H_2_O **+** β‐CD × 30) allowed us to further conclude that β‐CD addition during extraction provides better stability to phlorotannins when going through gastrointestinal digestion, which could be the result of a higher rate of host‐guest interactions formed during extraction, likely enhanced by microwave heating and by the prolonged time of contact. Additionally, MAE‐H_2_O‐β‐CD × 30 proved to be more effective in protecting phlorotannins than MAE‐EtOH **+** β‐CD × 30, further supporting its potential as a cleaner and greener approach to obtaining phlorotannin extracts with better stability.

These findings suggest that the β‐CD‐assisted method in aqueous microwave‐assisted extraction offers an environmentally friendly alternative to conventional solvent‐based phlorotannin extraction methods and contributes to enhanced bioaccessibility of the phlorotannins. By reducing the degradation occurring during digestion, this approach helps increase the amount of phlorotannins released and available for absorption at the final stages of the intestinal phase. The improvement in stability and bioaccessibility herein reported in vitro, if reproducible in vivo, is likely to translate into higher bioavailability. To confirm the extent of the systemic absorption, in vivo studies would be required. Future studies should explore the functional benefits of these extracts in biological systems, paving the way for their application in nutraceuticals and functional foods.

## Nomenclature

CD: cyclodextrin
DMBA: 2,4‐dimethoxybenzaldehyde
GI: gastrointestinal
MAE: microwave assisted extraction
MAE‐EtOH: ethanol microwave assisted extract
MAE‐EtOH **+** β‐CD: ethanol microwave assisted extract followed by β‐cyclodextrin addition
MAE‐H_2_O: water microwave assisted extract
MAE‐H_2_O‐β‐CD: water microwave assisted extract with β‐cyclodextrin
MAE‐H_2_O **+** β‐CD: water microwave assisted extract followed by β‐cyclodextrin addition
MAE‐H_2_O‐γ‐CD: water microwave assisted extract with γ‐cyclodextrin
PhGE: phloroglucinol equivalents
TPhC: total phlorotannin content

## Author Contributions

**Sofia F. Reis**: conceptualization, methodology, formal analysis, validation, data curation, writing – original draft, writing – review and editing, visualization. **Marcelo D. Catarino**: conceptualization, methodology, data curation, formal analysis, validation, writing – original draft, writing – review and editing, visualization. **Susana S. Braga**: methodology, validation, investigation, formal analysis, data curation, writing – review and editing. **Manuel A. Coimbra**: validation, resources, writing – review and editing. **Artur M.S. Silva**: validation, resources, funding acquisition, writing – review and editing. **Susana M. Cardoso**: project administration, supervision, resources, validation, writing – review and editing.

## Conflicts of Interest

The authors declare no conflicts of interest.

## Supporting information



Table S1. Experimental design and corresponding response values for MAE of *Fucus vesiculosos* with water.

Table S2. Optimum points selected by the models or combination of the models.
