# Supplementary material for: Improving in vitro Gastrointestinal Stability of Phlorotannins From Food Grade Fucus vesiculosus Extracts Using Cyclodextrins
Source: J Food Sci. 2026 Jan 13;91(1):e70830. doi: 10.1111/1750-3841.70830 (PMC12797003; doi:10.1111/1750-3841.70830)
Supplement: Supplementary file 1 — Table S1. Experimental design and corresponding response values for MAE of Fucus vesiculosos with water. [file JFDS-91-0-s001.docx]

**Table S1.** Experimental design and corresponding response values for MAE of *Fucus vesiculosos* with water.

| **Assay** | **Fucus (g)** | **H_2_O (mL)** | **Ratio**  **(g/mL)** | | **Temperature (ºC)** | | **Time (min)** | |  | **Yield (%)** |  | **mg PhG/ g DW extract** |  | **mg PhG/ g DW algae** |
| --- | --- | --- | --- | --- | --- | --- | --- | --- | --- | --- | --- | --- | --- | --- |
| 1 | 1.1 | 60 | 0.018 | *(0)* | 100 | *(0)* | 11 | *(2)* |  | 39.5 |  | 2.7 |  | 1.07 |
| 2 | 1.1 | 60 | 0.018 | *(0)* | 100 | *(0)* | 7 | *(0)* |  | 39.5 |  | 3.0 |  | 1.10 |
| 3 | 1.5 | 60 | 0.025 | *(1)* | 150 | *(1)* | 10 | *(1)* |  | 57.7 |  | 1.3 |  | 0.74 |
| 4 | 1.5 | 60 | 0.025 | *(1)* | 50 | *(-1)* | 3 | *(-1)* |  | 26.9 |  | 2.7 |  | 0.72 |
| 5 | 0.6 | 60 | 0.010 | *(-1)* | 50 | *(-1)* | 3 | *(-1)* |  | 36.9 |  | 2.7 |  | 0.99 |
| 6 | 1.1 | 60 | 0.018 | *(0)* | 100 | *(0)* | 2 | *(-2)* |  | 39.0 |  | 2.5 |  | 1.00 |
| 7 | 0.6 | 60 | 0.010 | *(-1)* | 150 | *(1)* | 10 | *(1)* |  | 59.3 |  | 1.2 |  | 0.72 |
| 8 | 1.6 | 60 | 0.027 | *(2)* | 100 | *(0)* | 7 | *(0)* |  | 38.6 |  | 1.8 |  | 0.71 |
| 9 | 1.1 | 60 | 0.018 | *(0)* | 100 | *(0)* | 7 | *(0)* |  | 40.0 |  | 2.2 |  | 0.99 |
| 10 | 0.5 | 60 | 0.008 | *(-2)* | 100 | *(0)* | 7 | *(0)* |  | 41.5 |  | 2.3 |  | 0.97 |
| 11 | 1.5 | 60 | 0.025 | *(1)* | 50 | *(-1)* | 10 | *(1)* |  | 24.3 |  | 2.3 |  | 0.56 |
| 12 | 0.6 | 60 | 0.010 | *(-1)* | 50 | *(-1)* | 10 | *(1)* |  | 37.3 |  | 3.2 |  | 1.19 |
| 13 | 1.5 | 60 | 0.025 | *(1)* | 150 | *(1)* | 3 | *(-1)* |  | 53.2 |  | 1.5 |  | 0.82 |
| 14 | 1.1 | 60 | 0.018 | *(0)* | 36 | *(-2)* | 7 | *(0)* |  | 30.0 |  | 2.6 |  | 0.79 |
| 15 | 0.6 | 60 | 0.010 | *(-1)* | 150 | *(1)* | 3 | *(-1)* |  | 54.9 |  | 1.8 |  | 0.92 |
| 16 | 1.1 | 60 | 0.018 | *(0)* | 164 | *(2)* | 7 | *(0)* |  | 60.3 |  | 0.3 |  | 0.20 |

^PhG – phloroglucinol equivalents; DW – dry weight.^
