# Supplementary material for: Improving in vitro Gastrointestinal Stability of Phlorotannins From Food Grade Fucus vesiculosus Extracts Using Cyclodextrins
Source: J Food Sci. 2026 Jan 13;91(1):e70830. doi: 10.1111/1750-3841.70830 (PMC12797003; doi:10.1111/1750-3841.70830)
Supplement: Supplementary file 2 — Table S2. Optimum points selected by the models or combination of the models. [file JFDS-91-0-s002.docx]

**Table S2.** Optimum points selected by the models or combination of the models.

|  | **Ratio**  **(g/mL)** | **Temperature (ºC)** | **Time (min)** |  | **Yield (%)** | **mg PhG/ g DW extract** | **mg PhG/ g DW algae** |
| --- | --- | --- | --- | --- | --- | --- | --- |
| Yield | 1:100 | 150 | 7 |  | 56 ± 3 | 1.67 ± 0.4 | 0.63 ± 0.2 |
| TPhC (extract) | 1:100 | 66 | 7 |  | 38 ± 2 | 4.34 ± 1.0 | 1.08 ± 0.3 |
| TPhC (algae) | 1:100 | 84 | 3 |  | 40 ± 3 | 4.09 ± 0.9 | 1.17 ± 0.3 |
| Yield & extract | 1:100 | 85 | 7 |  | 40 ± 2 | 4.20 ± 0.9 | 1.09 ± 0.4 |
| Yield & algae | 1:100 | 127 | 3 |  | 47 ± 3 | 2.94 ± 0.7 | 1.00 ± 0.3 |
|  |  |  |  |  |  |  |  |
| Chosen | 1:100 | 130 | 3 |  | 48 ± 3 | 2.81 ± 0.6 | 0.98 ± 0.3 |

^TPhC- total phlorotannins content; PhG- phloroglucinol equivalents^
